# Ameliorated Mechanical and Dielectric Properties of Heat-Resistant Radome Cyanate Composites

**DOI:** 10.3390/molecules25143117

**Published:** 2020-07-08

**Authors:** Hou-Yu Li, Chang-Ming Li, Jun-Guo Gao, Wei-Feng Sun

**Affiliations:** Key Laboratory of Engineering Dielectrics and Its Application, Ministry of Education, School of Electrical and Electronic Engineering, Harbin University of Science and Technology, Harbin 150080, China; houyu_li@126.com (H.-Y.L.); kingstel@163.com (C.-M.L.); gaojunguo@hrbust.edu.cn (J.-G.G.)

**Keywords:** cyanate ester, break elongation, dielectric permittivity, glass transition, radome material

## Abstract

In order to improve the mechanical and dielectric properties of radome cyanate, a synergistic reinforcement method is employed to develop a resin-based ternary-composite with high heat-resistance and preferable radar-band transmission, which is expected to be applied to fabricate radomes capable of resisting high temperature and strong electric field. According to copolymerization characteristics and self-curing mechanism, epoxy resin (EP) and bismaleimide (BMI) are employed as reinforcements mixed into a cyanate ester (CE) matrix to prepare CE/BMI/EP composites of a heat-resistant radome material by high-temperature viscous-flow blending methods under the catalysis of aluminum acetylpyruvate. The crystallization temperature, transition heat, and reaction rate of cured polymers were tested to analyze heat-resistance characteristics and evaluate material synthesis processes. Scanning electron microscopy was used to characterize the micro-morphology of tensile fracture, which was combined with the tensile strength test and dynamic thermomechanical analysis to investigate the composite modifications on tenacity and rigidity. Weibull statistics were performed to analyze the experimental results of the dielectric breakdown field, and the dielectric-polarization and wave-transmission performances were investigated according to alternative current dielectric spectra. Compared with the pure CE and the CE composites individually reinforced by EP or BMI, the CE/BMI/EP composite acquires the most significant amelioration in both the mechanical and electrical insulation performances as indicated by the breaking elongation and dielectric breakdown strength being simultaneously improved by 40%, which are consistently manifested by the obviously increased transverse lines uniformly distributed on the fracture cross-section. Furthermore, the glass-transition temperature of CE/BMI/EP composite reaches the highest values of nearly 300 °C, with the relative dielectric constant and dielectric loss being mostly reduced to less than 3.2 and 0.01, respectively. The experimental results demonstrate that the CE/BMI/EP composite is a highly-qualified wave-transmission material with preferences in mechanical, thermostability, and electrical insulation performances, suggesting its prospective applications in low-frequency transmittance radomes.

## 1. Introduction

Transmittance materials are primarily applied in radome manufactures to ensure that the antenna device system operates smoothly in remote control and communications, resisting the mechanical, thermal, electrical, and chemical impacts in harsh environments. The stealth function required for recently progressive radomes has been achieved by coordinating the diversified characteristics of wave-absorption at high frequencies and wave-transmission at low frequencies, which is intriguing for developing next-generation transmittance materials [1,2,3]. At present, the wave-transmission materials used for fabricating radomes are mainly composed of ceramics and resins. It is difficult to densify and mold the ceramic radomes with an adequate tenacity in the sintering process normally used for preparing ceramic materials [4,5]. In comparison, resin-based transmittance materials are preferred for their higher tenacity in a sufficient temperature range, which can be obtained through a casting molding process used for radome fabrications [6,7].

Cyanate ester (CE) is a thermosetting resin polymer with relatively high thermal stability and a characteristic molecular structure containing two or more cyanate functional groups –(OCN). CE materials show minimal water absorption (<1.5%) and higher bending and tensile strengths than that of epoxy resin. The glass-transition of CE resin generally occurs at the temperature range of 240~300 °C, which can be increased to the maximum value of 400 °C after being thermally modified [8,9,10]. CE resin possesses an extremely low relative dielectric constant (2.8~3.2) and a neglectable dielectric loss tangent (0.002~0.008), which almost remain unchanged in the variations of temperature and wave-frequency. Therefore, CE resin with high thermal stability and low electromagnetic wave dispersion is a prospective resin-based candidate of broadband radome materials in the future [11,12]. However, the large numbers of aromatic rings in the CE molecular structure will lead to considerable brittleness and low toughness of the cured resin system and thereby cannot meet the high tenacity requirement for mechanically protective radomes [13,14,15]. Moreover, the substantial toxicity of raw materials for synthesizing CE resin restricts its sustainable applications [16]. With the original excellent properties being maintained, the deficient properties of CE resin materials can be improved by means of mixing multiple organic resins or introducing functional polar-groups into CE molecular chains [17,18,19].

Previous studies for developing CE-based modified materials have always concentrated on the improvement of an objective performance for radome applications by individually blending or combining specialized reinforcement agents to the CE matrix. These research schemes are incompetent at taking into account the comprehensive characteristics of the modified materials, which sets substantial limitations on the practical applications of CE resin. For instance, breakable chain segments produced from the group reactions of amino-terminal polypropylene and CE trimers can be embedded in the backbone of the cured network to decrease the crosslinking density and increase the tenacity of CE resin, which will however degrade electrical resistance and dielectric properties [6]; CE/carbon-fiber composites obtained through complex synthesis processes inherit the unique mechanical and heat-resistant characteristics of carbon fiber, however, they show a significantly decreased wave-transmittance due to the high dielectric permittivity of carbon fiber [20]. In the present paper, epoxy resin (EP) and bismaleimide (BMI) were synergistically exploited as a coordinate reinforcement to ameliorate the mechanical and electrical performances of CE transmittance materials. According to the self-curing principle, EP and BMI at a well acceptable content of 10 wt % with 0.3 wt % aluminum acetylpyruvate [21] as the catalysis for expediting the curing reactions were blended into the CE matrix and processed through the thermosetting reactions to develop a significantly modified transmittance material of ternary copolymer composite, which were experimentally verified by analyzing the mechanical, heat-resistant, and dielectric properties.

## 2. Experimental

### 2.1. Material Preparation

The fundamental raw materials used in the material synthesis experiments were as follows: white powdery crystals of bisphenol A cyanate ester (BACE, Hubei Jusheng Co. Ltd., Wuhan, China) as the monomer matrix; viscous liquid of epoxy resin monomer (E51, Luohe Advanced Material Co. Ltd., Shanghai, China) as a reinforcement agent; light yellow crystalline powder of bismaleimide monomer (BMI monomer, Guandao Chemistry Co. Ltd., Shanghai, China) as a coordination reinforcement agent; and white powder of aluminum acetylpyruvate (AA, Yuancheng Technology Development Co. Ltd., Wuhan, China) as the resin-curing catalysis. 

Reinforcement agent in 10% mass percentage (10 wt%) was adopted to individually prepare the CE-based composites modified with EP (CE/EP), BMI (CE/BMI) and EP together with BMI (CE/BMI/EP), as shown in Table 1, which lists the blending proportions of pristine materials for preparing CE–based copolymer composites. As reference, pure CE resin material was also obtained through the identical curing process as that used for the composites. The oven was heated up to a temperature of 40 °C, persisting for 10 min under evacuation, and then put the forming mold coated with a mold-releasing agent into the oven and heated up to 60 °C to preheat for 20 min. The heated mixing and curing processes of synthesizing resin composites were carried out with an oil bath pan: the monomer matrix material (BACE) was put into the beaker container in the oil bath pan and heated up to melt, then the reagents of auxiliary materials (E51 and BMI monomer) and 0.3 wt % catalyst (AA) were added to be evenly stirred for 10 min, leading to a uniformly blended melt that was subsequently poured into the preheated mold in the oven being evacuated to vacuum; reaction temperatures of the curing processes are controlled by a step-heating method as 140 °C × 1 h + 160 °C × 2 h + 180 °C × 2 h + 200 °C × 3 h + 230 °C × 3 h and naturally cooled down to ambient temperature. The casting samples were finally achieved after demolding.

**Figure 1:**
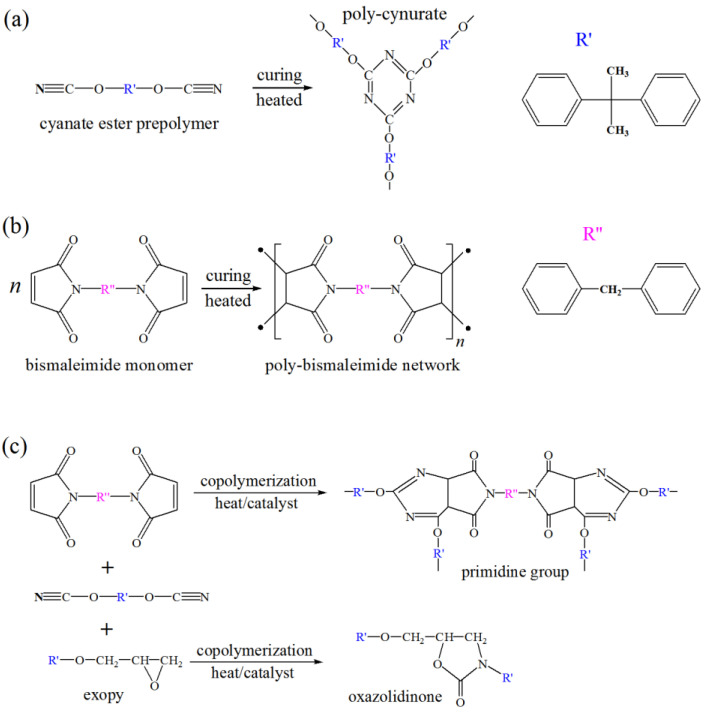
Schematic polymerization of the blended CE, BMI and EP monomers. (**a**) CE self-polymerization; (**b**) BMI self-polymerization; (**c**) copolymerization of CE with BMI and EP.

Curing reaction of the CE monomers (pre-polymers) with reactive (–O–C≡N) functional groups is addition polymerization without producing any by-products, in which the ring trimerizations will occur to form a three-dimensional cross-linked network when being heated, as shown in Figure 1a, which schematically illustrates CE self-polymerization.

BMI monomers also undergo self-polymerization when being heated, with the hydroxyl in one imide ring as the electron donor and the electron-deficient double-bond in another imide as the electron acceptor, in which two independent radicals are produced by the formation of electron donor-to-acceptor complex to initiate the chain growth reactions, as schematically shown in Figure 1b for BMI self-polymerization. The polymerization reactions for the ternary EP/BMI/CE composite are complicated to generate various products, among which the curing reaction of EP is usually accompanied with the curing agents to initiate the reactions, otherwise the BMI and EP can dissolve each other in any proportion to be well blended into CE. In the curing process under gradient-heating, the reaction products are dominated by the oxazolidinone organic structure and heterocyclic pyrimidine groups [22,23], as shown in Figure 1c for the schematic polymerization mechanism of BMI and EP blending with CE.

### 2.2. Material Characterization and Property Test

In order to characterize the fractured micro-structures related to mechanical properties, the cross-sections of the quick-frozen brittle fractures, which had been obtained by treating the demolded sheet samples of 30 mm × 30 mm × 1 mm in liquid nitrogen, were observed by an ultra-high-resolution cold field emission scanning electron microscope (SU8020, Hitachi Co. Ltd., Tokyo, Japan) with a magnification of 2k under the accelerating voltage of 0.5 kV~30 kV. The tensile stress-strain characteristics, by which the tensile strength, Young modulus, and breaking elongation can be evaluated, were measured according to the GB/1447-2005 standard with a mechanical elongation testing machine (QX-W300, Shanghai Qixiang Test Instruments Co. Ltd., Shanghai, China) to estimate the material tenacity improved by the composite modifications. By employing the dynamic mechanics analysis (DMA) method, the viscoelasticity of the resin-based composites is evaluated by measuring the energy-storage modulus at different temperatures as implemented on a dynamic thermomechanical analyzer (Q800DMA, TA apparatus Co. Ltd., Delaware, USA) under a nitrogen atmosphere with the samples being scaled as 50 mm × 10 mm × 1 mm and a heating rate of 5 °C/min at the testing temperature range of 30~300 °C.

According to the differential scanning calorimetry (DSC) method, the variation curves of heat-flow vs. temperature before and after the resin curing process were tested using a thermal analyzer (DSC-3, METTLER TOLEDO, Zurich, Switzerland) under a nitrogen atmosphere at the temperature range of 30~300 °C with a heating rate of 10 °C/min. The exothermic-peak and glass-transition temperatures were evaluated to investigate the curing process and heat-resistant characteristics of the prepared materials.

Direct current (DC) electrical insulation performances of the circular film samples with a diameter of 80 mm and a thickness of 0.1 mm were tested with the film breakdown strength tester (BTF-072, Changchun Peterford Technology Co. Ltd., Changchun, China) by measuring the dielectric breakdown voltage according to the ASDTM149 standard, in which the voltage-increasing rate and dielectric capacitance are controlled to 500 V/s and 6 kV·A, respectively. The tested results of the dielectric breakdown field were analyzed though Weibull 2-parameter statistics. Alternative current (AC) dielectric polarization behaviors (complex dielectric permittivity spectrum) of the circular film samples with both sides being vacuum evaporated by aluminum film electrodes with a 25 mm diameter were tested in a scanned frequency range of 10 kHz~10 GHz at ambient temperature utilizing a wide-frequency dielectric spectrometer (Alpha-A, Novocontrol Co. Ltd., Frankfurt/Main, Germany) to obtain the dielectric constant and loss spectra in a wave-region comprising x-band (radar band), by which the dielectric properties and wave-transmittance of CE-resin composites are studied.

## 3. Results and Discussion

### 3.1. Micro-Morphology Characterization

**Figure 2:**
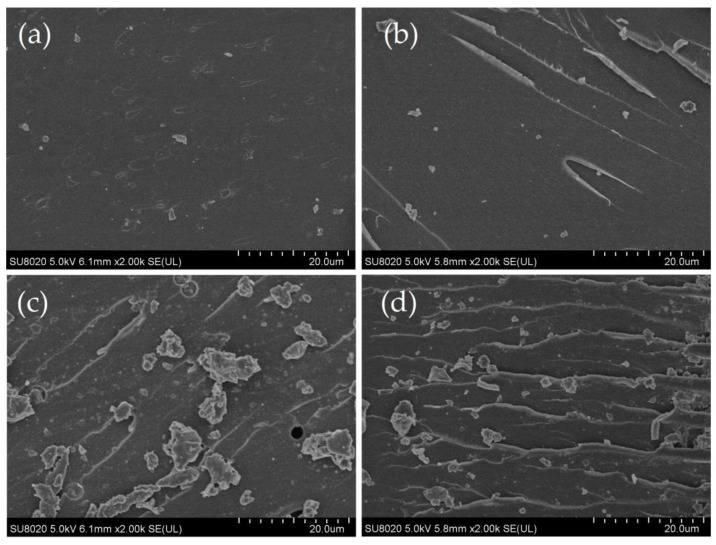
SEM images of brittle-fractured cross-sections of (**a**) CE, (**b**) CE/BMI, (**c**) CE/EP, and (**d**) CE/BMI/EP.

The fracture cross-sections of pure CE, CE/BMI, CE/EP, and CE/BMI/EP composites after being cured were characterized by SEM, as seen in Figure 2. It can be seen in the SEM images that no observable phase separation arise in all four resin materials, implying good miscibility between the blended components. The relatively smooth fracture surface of pure CE represents a typical brittle fracture. The fracture surface of the CE/BMI composite appears to have a few ductile-vortex grooves with long crack lines, and the bottom of the groove is flat, while the fracture of CE/EP composite shows an increment of ductile-vortex grooves with short cracks. In comparison, the resin fracture of the CE/BMI/EP composites exhibit dense and uniformly aligned striations of teared layers, indicating a typical ductile fracture. 

It is indicated from Figure 2b that the CE/BMI copolymer composite has undertaken a complete copolymerization reaction to form a homogeneous structure. Whereas due to insufficient copolymerization, the separated phase of incompatible homopolymer arises from the self-polymerization of CE monomers and EP fortifiers, as indicated by dispersed fragments on the fractured cross-section of the CE/EP copolymer composite in Figure 2c. In contrast, the smaller and fewer fragments in Figure 2d clearly illustrates the significant attenuation of homopolymer phase separation, caused by the more sufficient ternary copolymerization of the CE/BMI/EP copolymer composite. Therefore, the EP/BMI synergistic reinforcement can evidently promote the copolymerization reaction for CE-resin composite modifications, which will effectively increase material toughness.

### 3.2. Mechanical Property

**Figure 3:**
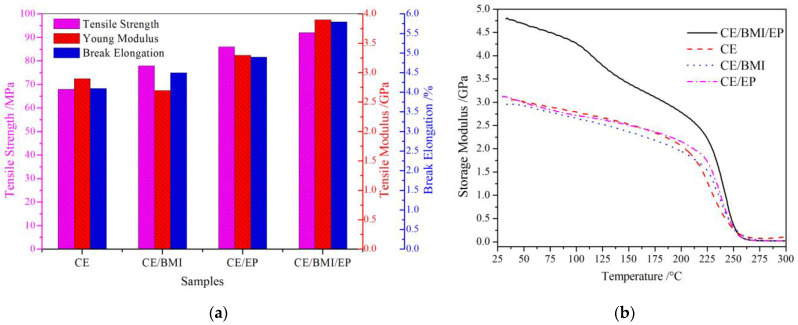
(**a**) The tensile strength, Young modulus, and break elongation of pure CE and CE-based composites; (**b**) DMA temperature spectra of pure CE and CE/EP, CE/BMI, and CE/BMI/EP composites after curing.

The tensile strength, Young modulus, and break elongation of the CE resin and composite materials were evaluated from the elongation stress-strain tests with the results being shown in Figure 3a. After being blended with BMI and EP reinforcements in the curing process, the prepared CE-based composites show conspicuous enhancements in both tensile strength and break elongation. Even though the CE/BMI and CE/EP composites, respectively, show lower and higher tensile elastic modulus than that of pure CE, and the CE/BMI/EP composite acquires the highest value due to the synergistic BMI and EP reinforcements. The CE/BMI/EP composite represents the most distinctive improvement in tensile tenacity with a break elongation increment of 30% compared with CE resin, due to the complete curing reactions of synergistic reinforcements. Cyanate and epoxy groups are fairly compatible to be dissolved in any proportion and partially react to form oxazoquinolinone organic structures with a substantially higher modulus, leading to stronger intermolecular forces and thereby showing a higher elastic modulus under external mechanical loading [24]. A considerable number of reticular triazine ring structures generated by heating self-polymerization in the cured CE resin have been expanded and entangled by the BMI homopolymers, accounting for the reduced brittleness and increased tensile strength of the composites [25].

The DMA testing results of pure CE and CE-based composites are shown in Figure 3b. The CE/BMI/EP composite acquires remarkably higher storage modulus at temperatures from 20 °C to 200 °C, indicating that the increased intermolecular forces limit the movement of molecular chain segments and a more stable molecular morphology has been achieved by a coordinated reinforcement of BMI and EP. At the temperatures of 200~270 °C when approaching the glass-transition temperature (*T*_g_), the transformation of molecular-chain motions from localized vibrations to random global displacements results in the nosedive ability to store elastic deformation energy [26,27]. After the temperature reached *T*_g_ ~ 270 °C, the polymer molecules of all the three composites will exist in a loose or free aggregation state when elastic energy storage promptly tends to almost zero [28].

With the combination of SEM micro-morphology, mechanical tensile experiment, and DMA analysis, it can be reasonably summarized that the fully blended and polymerized (after curing) CE/BMI/EP composite with a tight molecular aggregation possesses excellent mechanical properties including the quintessential ductile fracture and high tenacity. Therefore, we have successfully developed a deformation-stable CE/BMI/EP composite with significantly improved performances of tensile resistance and elastic energy storage.

### 3.3. Thermal Characteristics

**Figure 4:**
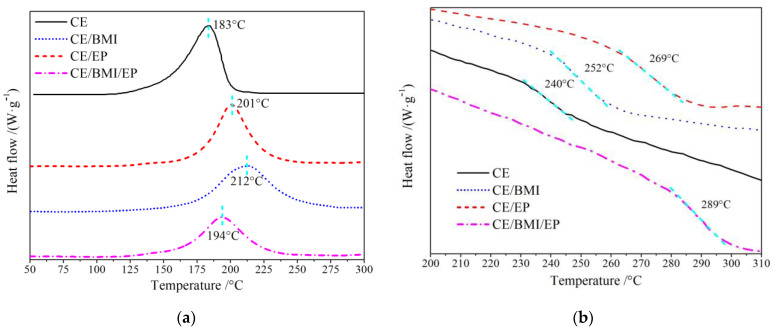
Dynamic differential scanning calorimetry (DSC) exothermic temperature spectra: (**a**) in curing process and (**b**) glass transition of the cured materials for the pure CE and CE/EP, CE/BMI, CE/BMI/EP composites.

Curing temperatures of CE resin and its composites were measured employing DSC dynamic tests performed in resin-curing processes, as per the results shown in Figure 4a. The curing temperature can be determined directly by the location of the characteristic exothermic peak in the dynamic DSC spectrum for the curing process. The CE prepolymers can be cured by one specific self-polymerization under the lowest temperature of 183 °C. After being modified by EP or/and BMI reinforcements, the peak curing temperature is increased by 10~30 °C in reference to pure CE, in which the CE/BMI/EP composite shows a significantly lower curing temperature of 194 °C than the other binary composites, further confirming the synergistic effect of simultaneous BMI and EP modification that is beneficial to curing tractability. Aside from the alike peak curing temperature like pure CE, the CE/BMI/EP composite shows a wider temperature range around the DSC peak, implying that the polymerization reactions are facilitated in a milder curing process and more suitable for casting. The curing reaction of CE/BMI/EP composite consists of CE self-polymerization, CE copolymerization with EP or BMI, and BMI self-polymerization [29,30], as schematically shown in Figure 1c.

Glass-transition temperature *T*_g_ is an essential thermal property of resin materials, which indicates the thermal stability and determines the working temperature range in thermodynamic environments. The cured materials of CE and modified composites were also tested for the DSC temperature spectra by which *T*_g_ can be effectively evaluated, as seen in the results shown in Figure 4b, which is well consistent with the DMA results of Figure 4. The structural morphology of cured CE transforms at 240 °C (*T*_g_), which is lower than the CE-based composites due to the mixture effects on the melting point of a multiple-phase system. Otherwise, the ternary CE/BMI/EP composite shows an approximate *T*_g_ of 289 °C, which is definitely higher than the other binary composites, implying that the preferred enhancement of heat-resistance has been achieved in concomitance with mechanical modification.

The polymerization structures of the ternary composite obtained by simultaneous EP and BMI modification in curing reaction process were more complex than the other resin materials, leading to the qualitative changes in polymer aggregation. The self-polymerization reaction occurs in the CE monomer matrix after being heated to curing temperature to form the regularly reticulated configurations of densely distributed triazine-rings, which are capable of restricting the molecular segments to a stable state below a specific high temperature [31], whereas EP or BMI reinforcement will abate the regularity of CE reticulated configurations and thus reduce the intermolecular forces, resulting in a decreased kinetic energy needed for molecular chains to escape from van der Waals interactions at a lower *T*_g_. Nevertheless, in the extended curing process of the CE/BMI/EP composite, the copolymerizations of EP and BMI with CE monomers can produce various heterocyclic derivatives of pyrimidine, which will substantially intensify intermolecular entanglements. Meanwhile, the formation of CE network structures is expedited by BMI reinforcement with a minority of BMI self-polymerization reactions, which will affirmatively improve the crosslinking density of curing networks and the aggregation of polymer products. Therefore, a larger kinetic energy of molecular segments is required to destroy the polymeric aggregating configurations for the qualitative transformation from the elastomeric state to amorphous glassy morphology. Moreover, the more flexible oxazolidone groups will be generated from the reactions of EP with CE monomers, which hinders the segment movements of molecule chains and will raise material viscosity. Accordingly, a higher temperature is required for qualitatively exacerbating molecule vibrations to break the viscous resistance of the CE/BMI/EP composite, resulting in the evident inhibition on glass-transition and the excellent heat-resistance retained from the CE resin.

### 3.4. Electrical Performance

**Figure 5:**
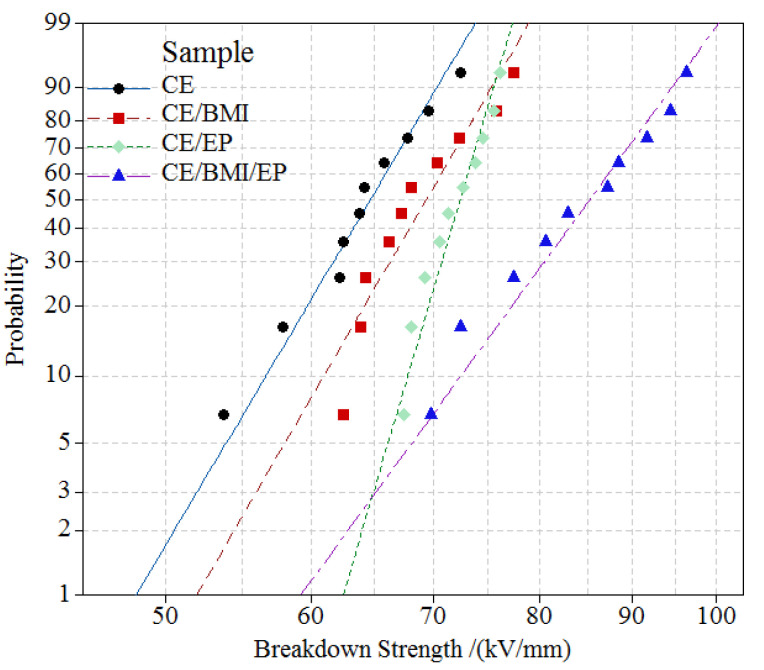
DBS fitted with 2-parameter Weibull statistics of pure CE and CE/EP, CE/BMI, and CE/BMI/EP composites.

Two-parameter Weibull Statistics was employed to analyze the dielectric breakdown strength (DBS) results being tested at ambient temperature, as shown in Figure 5. Scale-parameter represents the characteristic breakdown field at 63.2% probability, and shape-parameter indicates the dispersion of the breakdown data. The characteristic breakdown field *E*_b_ and shape-parameter *β* from the fitted Weibull distributions of DBS data are listed in Table 2. Compared with the CE resin, the CE/BMI/EP composite presented a 40% increment of *E*_b_ and 19% reduction of *β*, which indicate the remarkable improvements of DBS and high-voltage resistance stability, while the two binary composites show only 11% DBS enhancement with degraded high-voltage resistances. The polymerization reactions of the ternary composite in the curing process are more complicated than the pure CE resin and binary composites, which render the multiple heterocyclic structures including the oxazoquinolinone and pyrimidine produced by the CE copolymerizations with EP and BMI, respectively, leading to the abatement of intermolecular polarity and the amelioration of electrical insulation performance [32]. Meanwhile, the self-polymerization reactions of CE and BMI monomers form interpenetrating polymer network structures that can prevent the curing molecules from aggregating in a localized space and impede the space-charge accumulating on structural defects under external electric field, resulting in substantial improvements of DBS and insulation-stability. 

**Figure 6:**
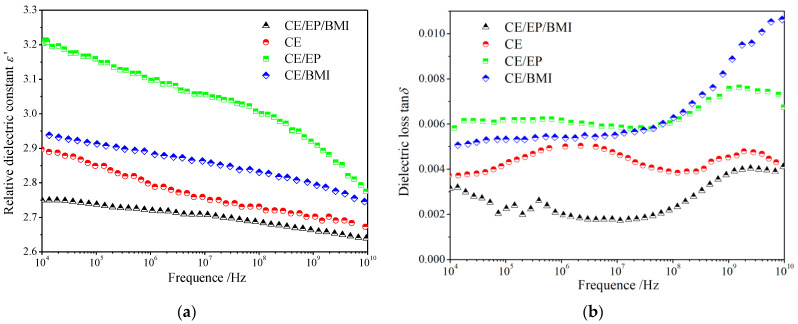
Complex dielectric functions of the pure CE and CE/EP, CE/BMI, and CE/BMI/EP composites: (**a**) relative dielectric constant and (**b**) dielectric loss tan*δ*.

Dielectric characteristics of wave-transmittance materials determine the electromagnetic-wave transmissivity and is the most important index to evaluate the performance of radome. The relative dielectric constants and dielectric loss of the pure CE and CE/EP, CE/BMI, and CE/BMI/EP composites as a function of AC electric field frequency are shown in Figure 6. The dielectric permittivities of the four materials all show downward trends in a wide frequency range of 10 kHz~10 GHz, in which the CE/BMI/EP composite is optimal with the lowest real dielectric spectrum between 2.6 and 2.8 in the smallest variation amplitude, which ensures obtaining an efficient and stable signal transmission in the X-band. The totally larger dielectric loss of the CE/EP composite increases monotonously with the rising frequency in contrast to the spectrum fluctuations of the other three materials, while the lowest dielectric loss of 0.002~0.004 has been acquired by the CE/BMI/EP composite in the whole testing frequency range. In particular, the CE/BMI/EP composite persists with a constant minimum value of dielectric loss in 10^6^~10^8^ Hz, which comprises a characteristic low frequency x-band of skywave over-the-horizon radar.

AC dielectric spectra indicate that the CE/BMI/EP composite also has earned a promotion in dielectric properties. The low dielectric polarization characteristics of CE resin originate from the polar configuration formed by self-polymerization. After the collaborative modifications of EP and BMI reinforcements, a new stable composite structure with smaller intermolecular voids has been achieved through a variety of copolymerization reactions, which restricts the movements of polar structures and obtains a faster dielectric response around intermolecular voids. In contrast, the dense reticulations of CE resin are partially interrupted by BMI or EP reinforcement to form larger intermolecular spaces and possibly cause two-phase interfaces in the binary composites, leading to a higher activity of polar molecule segments and the interfacial polarization with longer relaxations. Therefore, the synergistic effect of coordinate reinforcement is also valid for improving the dielectric performances of CE resin. Simultaneously, with a higher breakdown-resistance than CE, the low dielectric constant and loss of CE/BMI/EP composite determines the high-quality signal transmission coefficient and the high-efficiency electromagnetic wave transmittance in the radar wave-band, suggesting a preferable wave-transmittance material in prospective applications of antenna radomes. To this end, mechanical, thermal, and dielectric characteristics of the CE/BMI/EP composite are listed in Table 3 in comparison with other reports, which highlights the comprehensive competitiveness of the strategic CE-resin modifications represented in this paper.

## 4. Conclusions

A resin-based ternary-composite with high heat-resistance and preferable radar-band transmission has been developed to ameliorate the mechanical and dielectric properties of a cyanate copolymer used for antenna radomes. The synergistic reinforcement method is suggested to synthesize resin-based transmission materials being capable of resisting high temperature and strong electric field. The CE resin as a fair wave-transmittance material is significantly modified in terms of the mechanical and dielectric properties by a synergistical reinforcement scheme of blending EP and BMI into the CE matrix. The prepared materials have been systematically investigated by the micro-morphology SEM and mechanical elongation experiments in combination with dynamic thermomechanical analyses. The curing process and glass-transition are shown in the DSC tests, and the DC electrical insulation and wave-transmittance performances are evaluated through DBS Weibull statistics and AC complex dielectric spectra. The CE/BMI/EP composite achieved by coordinately blending BMI and EP reinforcements represents a preferable processing character of curing at the temperature of 194 °C with a wide and flat exothermic peak for easy casting. The cured CE/BMI/EP composite shows a higher glass-transition temperature with an improvement in heat-resistance compared with pure CE resin. The fractured surface of the CE/BMI/EP composite illustrates the evident ductile striations, and the tensile strength approaches 96 MPa with a break-elongation of 5.9%, implying that the intermolecular forces have been enhanced by simultaneously blending BMI and EP into the CE matrix to realize a compatibly copolymerized ternary composite. Meanwhile, the CE/BMI/EP composite acquires the considerable increments in mechanical energy-storage and stiffness, confirming the promoted tenacity observed by SEM. Compared with the binary-composite, the highly intensified intermolecular interactions in the ternary composite result in the reduction of free spaces for charge transports, as indicated by the DBS increment of 40%. The relative dielectric constant and dielectric loss of the CE/BMI/EP composite decline to 2.6~2.8 and 0.002~0.004, respectively, in a wide frequency range of 10 kHz~10 GHz, which means an extremely low dielectric polarization specified for high quality transmittance materials. In contrast to the CE resin, the CE/BMI/EP composite as an efficient wave-transmittance material possesses the analogical processability and higher heat-resistance as well as with the mechanical strength being significantly improved. In a wide X-band of radar communications, the CE/BMI/EP composite represents an evident reduction in dielectric permittivity and transmission loss in comparison to the CE resin, demonstrating a notably higher electromagnetic wave-transmittance. In brief, the CE/BMI/EP composite acquires an appreciable amelioration simultaneously in mechanical tensile strength, electrical insulation performance, and heat-resistance, promising its competency in applications for the radar radomes requiring for high mechanical strength and electrical insulation.

## Figures and Tables

**Table 1:** Composition of pristine materials for preparing the CE–based copolymer composites.

Samples	Mass Ratio (CE:BMI:EP)	Catalyst/wt%
CE	10:0:0	0
CE/BMI	10:1:0	0.3
CE/EP	10:0:1	0.3
CE/BMI/EP	10:1:1	0.3

**Table 2:** Weibull distribution fitted 2-parameters in 95% confidence interval from the tested dielectric breakdown fields: characteristic breakdown field *E*_b_ at 63.2% probability and shape parameter *β*.

	2-Parameter	
	*E*_b_/(kV/mm)	*β*
CE	66.33	14.38
CE/BMI	71.13	14.64
CE/EP	73.32	18.73
CE/BMI/EP	87.94	11.64

**Table 3:** Comprehensive characteristics of CE-resin composites for comparison.

Composites/Characteristics	AlPO_4_/CE [33]	PPCE/BMI [8]	CE/EP/LC [34]	CE/EP/PEK-C [35]	CE/BMI/HBPSi [28]	CE/BMI/EP Present
Tensile strength/MPa	90	72	62	86	82	92
Tensile modulus/GPa	3.6	–	3.6	3.5	3.0	3.8
Elongation at break/%	5.42	3.67	3.35	4.44	3.96	5.82
Curing temperature/°C	210	135	198	185	–	194
*T*_g_/°C	271	277	280	234	268	289
Relative dielectric constants (10 GHz)	3.05	3.10	2.74	–	2.97	2.64
Dielectric loss tan*δ* (10 GHz)	0.0065	0.0170	0.0049	–	0.0063	0.0040
Transmittance band (tan*δ* ≤ 0.01)	Ku	S	C	–	S	X

## References

[1] Wang G., Wang R., Fu G., Gao T.L., Fu C., Kuang H., Yang F., Jiao W.C., Hao L.F. (2015). Study on phenolphthalein poly(ether sulfone)-modified cyanate ester resin and epoxy resin blends. Polym. Eng. Sci..

[2] Khatavkar N., Balasubramanian K. (2016). Composite materials for supersonic aircraft radomes with ameliorated radio frequency transmission-A review. RSC Adv..

[3] Costa F., Monorchio A. (2012). A frequency selective radome with wideband absorbing properties. IEEE Trans. Antennas Propag..

[4] Dippong T., Levei E., Cadar O., Goga F., Borodi G., Barbu-Tudoran L. (2017). Thermal behavior of Co_x_Fe_3−x_O_4_/SiO_2_ nanocomposites obtained by a modified sol-gel method. J. Therm. Anal. Calorim..

[5] Dippong T., Levei E., Cadar O., Goga F., Toloman D., Borodi G. (2019). Thermal behavior of Ni, Co and Fe succinates embedded in silica matrix. J. Therm. Anal. Calorim..

[6] Ma J.X., Lei X.F., Wang Y., Sun Y. (2018). Toughening modification of cyanate ester with amino-terminated polyoxypropylene. Iran. Polym. J..

[7] Marieta C., Rio M.D., Harismendy I., Mondragon I. (2000). Effect of the cure temperature on the morphology of a cyanate ester resin modified with a thermoplastic: Characterization by atomic force microscopy. Eur. Polym. J..

[8] Wu G., Cheng Y., Xie Q., Liu C., Kou K.C., Zhuo L.H., Wang Y.Q. (2014). Synthesis of a bismaleimide/cyanate ester copolymer containing phenolphthalein functional group with excellent dielectric properties and thermally stable. J. Polym. Res..

[9] Shi H., Fang Z., Gu A., Tong L., Xu Z. (2007). Carboxyl-terminated butadiene-acrylonitrile rubber modified cyanate ester resin. J. Appl. Polym. Sci..

[10] Hillermeier R.W., Seferis J.C. (2000). Environmental effects on thermoplastic and elastomer toughened cyanate ester composite systems. J. Appl. Polym. Sci..

[11] Liang G., Ren P., Zhang Z., Lu T.L. (2006). Effect of the epoxy molecular weight on the properties of a cyanate ester/epoxy resin system. J. Appl. Polym. Sci..

[12] Feng Y., Fang Z., Gu A. (2004). Toughening of cyanate ester resin by carboxyl terminated nitrile rubber. Polym. Adv. Technol..

[13] Nakamura S., Fujii T., Matsukawa S., Masayuki K., Fukuyama H. (2018). Specific heat, thermal conductivity, and magnetic susceptibility of cyanate ester resins—An alternative to commonly used epoxy resins. Cryogenics.

[14] He S., Liang G., Yan H., Wang J., Yang L. (2009). High performance toughened cyanate ester resin with low injection temperature for RTM process. Polym. Adv. Technol..

[15] Cao H., Liu B., Ye Y., Liu Y., Li P. (2019). Study on the relationships between microscopic cross-linked network structure and properties of cyanate ester self-reinforced composites. Polymers.

[16] Brahmbhatt P., Unnikrishnan J., Sudha J.D., Pradhan S. (2012). Cure kinetics studies of cyanate ester and bisphenol-F epoxy blend. J. Appl. Polym. Sci..

[17] Gu A. (2006). High performance bismaleimide/cyanate ester hybrid polymer networks with excellent dielectric properties. Compos. Sci. Technol..

[18] Chuang W., Geng S.J., Bao L.Z., Lei P., Wen M.H., Li P.Z. (2017). Modification of cyanate resin by conjugated tri-component interpenetrating polymer networks. J. Mater. Res. Technol..

[19] Suman N.J. (2005). Assessment of bismaleimide-modified cyanate ester as matrix resin for elevated service temperature carbon composite applications. J. Reinf. Plast. Compos..

[20] Ou Q.R., Ji P.J., Xiao J., Wu L. (2019). Study on the properties of resin transfer molding cyanate ester and its T800 grade carbon fiber composites. Fluid Dyn. Mater. Process..

[21] Hamerton I., Herman H., Mudhar A.K., Chaplin A., Shaw S.J. (2002). Multivariate analysis of spectra of cyanate ester/bismaleimide blends and correlations with properties. Polymer.

[22] Ravi S., Kishore S. (2007). Cure behavior of epoxy-cyanate ester blend in composite systems: Evaluation studies in neat resin cast by thermal and FTIR techniques. J. Appl. Polym. Sci..

[23] Wu F., Song B., Moon K.S., Wong C. Cyanate ester/epoxy co-curing system with thermal stabilizers for high temperature stability. Proceedings of the IEEE 68th Electronic Components and Technology Conference.

[24] Pradhan S., Brahmbhatt P., Sudha J.D., Unnikrishnan J. (2011). Influence of manganese acetyl acetonate on the cure-kinetic parameters of cyanate ester epoxy blend systems in fusion relevant magnets winding packs. J. Therm. Anal. Calorim..

[25] Hu X., Fan J., Yue C.Y. (2001). Rheological study of crosslinking and gelation in bismaleimide/cyanate ester interpenetrating polymer network. J. Appl. Polym. Sci..

[26] Wang M., Wei L., Zhao T. (2005). A novel condensation-addition-type phenolic resin (MPN): Synthesis, characterization and evaluation as matrix of composites. Polymer.

[27] Sudha J.D., Pradhan S., Viswanath H., Brahmb H.P., Manju M.S. (2014). Studies on the cure parameters of cyanate ester–epoxy blend system through rheological property measurements. J. Therm. Anal. Calorim..

[28] Guan Q., Gu A., Liang G., Li Y., Liao F., Gong Y.W. (2011). Curing kinetics and mechanism of novel high performance hyperbranched polysiloxane/bismaleimide/cyanate ester resins for resin transfer molding. J. Appl. Polym. Sci..

[29] Lin C.H., Yang K.Z., Leu T.S., Lin C.H., Sie J.W. (2006). Synthesis, characterization, and properties of novel epoxy resins and cyanate esters. J. Polym. Sci. Part A Polym. Chem..

[30] Wang W., Yuan L., Liang G., Gu A.J., Wu J.Y. (2010). Preparation and characterization of novel cyanate ester/epoxy resin microspheres. Colloid Polym. Sci..

[31] Chidambaram V., Rong E.P.J., Lip G.C., Daniel R.M.W. (2013). Cyanate ester-based encapsulation material for high-temperature applications. J. Electron. Mater..

[32] Liang G., Zhang M. (2002). Enhancement of processability of cyanate ester resin via copolymerization with epoxy resin. J. Appl. Polym. Sci..

[33] Sun Z., Huang P., Gu A., Liang G., Yuan L., Dai S. (2011). Novel high-performance wave-transparent aluminum phosphate/cyanate ester composites. J. Appl. Polym. Sci..

[34] Yu Y., Gan W., Liu X., Li S. (2010). Liquid crystalline epoxy resin modified cyanate ester/epoxy resin systems. J. Appl. Polym. Sci..

[35] Li J., Chen P., Ma Z., Ma K., Wang B. (2009). Reaction kinetics and thermal properties of cyanate ester-cured epoxy resin with phenolphthalein poly(ether ketone). J. Appl. Polym. Sci..

